# Effects of Carbon Doping and DC Bias Voltage on Microstructure and Mechanical Properties of AlCrCN Films Synthesized via HiPIMS

**DOI:** 10.3390/ma15165729

**Published:** 2022-08-19

**Authors:** Jian-Fu Tang, Shang-Hao Wang, Fu-Chi Yang, Chi-Lung Chang

**Affiliations:** 1 Department of Electronic Engineering, Lunghwa University of Science and Technology, Tao-Yuan 333326, Taiwan; 2 Department of Materials Engineering, Ming Chi University of Technology, New Taipei City 24301, Taiwan; 3 Center for Plasma and Thin Film Technologies, Ming Chi University of Technology, New Taipei City 24301, Taiwan

**Keywords:** high-power impulse magnetron sputtering, AlCrN, AlCrCN, mechanical properties, microstructure

## Abstract

This work compares the hardness and adhesion properties of AlCrN and AlCrCN hard coatings synthesized via HiPIMS using Al_70_Cr_30_ and Cr targets. The hardness and adhesion properties of AlCrCN films were optimized by performing deposition under various C_2_H_2_ flow rates (5, 8, 10, 13, 15, or 20 sccm) and DC bias voltages (−40, −60, −80, −100, or −120 V). EPMA results clearly indicated that the carbon content was increased from 1.9 to 12.2 at.% with increasing C_2_H_2_ flow rate from 5 to 20 sccm. XPS results confirmed a various content of chemical bonds (Cr-N, C-N, sp^2^, and sp^3^) with various C_2_H_2_ flow rate. Grain and columnar refinement in AlCrCN were derived from XRD, TEM, and SAED results. The higher hardness (28.6 GPa) and Young’s modulus (358 GPa) were obtained using an C_2_H_2_ flow rate of 5 sccm and a bias voltage of −60 V. Both of which subsequently decreased to 13.5 GPa and 212 GPa, respectively. This can be attributed to the C-N bond inhibiting the development of metal-N bonds. Increasing the bias voltage to −120 V increased the hardness to 32.9 GPa and the Young’s modulus to 372 GPa. Note that the application of bias voltage to enhance hardness should also be applicable to carbon-doped AlCrN films as well. All samples presented good adhesion characteristics (class 1; ISO26443:2008-06).

## 1. Introduction

According to Rizzo et al. [1], tools used for primary machining (e.g., milling, turning, and drilling) account for 87% of the overall tool market. During the machining process, interactions among the tool, workpiece, and ambient environment can lead to failure under the effects of wear, delamination, oxidation, and diffusion. The deposition of hard films can increase the life of cutting tools and workpiece without the prohibitive costs of developing new alloys. Over the last two decades, researchers have expended considerable effort in the development of transition metal nitride films (e.g., TiN, CrN, and ZrN) to extend the lifespan of cutting tools and workpiece. Researchers have yet to develop binary transition metal nitride films with the oxidation resistance required for modern industrial applications [2].

Researchers have demonstrated the superiority of ternary transition metal nitride coatings over their binary counterparts [3,4,5]. The hardness and thermal stability of AlCrN can be enhanced by making adjustments to metastable Al_x_Cr_1-x_N solid solutions and nanoscale domains [6]. The thermal stability of CrAlN films against oxidation (reaching 1000 °C) far exceeds that of binary nitride coatings (500–600 °C) [7,8,9]. AlCrN films present two distinct structures (FCC and HCP), which can be selected by altering Al doping content. A high Al content (e.g., >70 at.%) [10] can impose a phase transition from FCC to HCP, due to the solid solubility of Al atoms in CrN [11]. Note however that the hardness and wear resistance of HCP structure-AlN (Microhardness: 2 × 10^4^ N/mm^2^, abrasive wear rate: 8.3 m^3^/mN 10^15^) cannot match those of FCC structure-CrN (Microhardness: 2.8 × 10^4^ N/mm^2^, abrasive wear rate: 2.2 m^3^/mN 10^15^) [12]. We obtained similar results in a previous report [13,14], in which the mechanical properties of the Cr_70_Al_30_N coating (hardness: 33.6 GPa) were superior to those of a Cr_50_Al_50_N coating (hardness: 29.3 GPa). The hardness and wear resistance of those samples can be attributed to high Al content and an FCC structure. There has been little research on Al_30_Cr_70_N coatings of lower hardness.

CrCN coatings are used for protective coatings in marine environments [15,16], anti-wear coatings in joints [17], and anti-friction coatings in mechanical devices [18], due largely to their excellent properties at high temperatures [19]. The nanocrystalline/amorphous structure and hard Cr_7_C_3_ phase that forms when CrN coatings are doped with carbon can significantly improve the mechanical properties [20]. AlCN coatings have attracted considerable interest for their wide band gap, high chemical stability [21,22,23], and high hardness (30 GPa for AlCN coatings [24] and 18 GPa for AlN films) [25]. Tillmann [26,27] et al. analyzed the effects of the tribological performance of AlCrCN coatings with different C contents and bias voltages by DC/HiPIMS technologies. High-power impulse magnetron sputtering makes it possible to create thin films with density, hardness, and surface smoothness superior to those of conventional PVD technologies [28]. In the current study, our primary objective was to enhance the hardness of the hard coatings on cutting tools by creating Al_30_Cr_70_CN films via HiPIMS using targets of Al_70_Cr_30_ and Cr target with a focus on the effects of C_2_H_2_ flow ratio and bias voltage.

## 2. Experiment Details

Coating was performed in a commercial-grade deposition chamber (YG-1009-HL, Taiwan) connected to four pulsed power sources (Hüttinger 4002 G2, Ditzingen, Germany) with two Cr and two Al_70_Cr_30_ targets (dimensions: 49 × 9 cm). The structure of the coating in the current study was as follows: AlCrCN/AlCrN/Cr on a tungsten carbide substrate. All tungsten carbide substrates (WC, 92 at.%; Co, 8 at.%; diameter, 25 mm; thickness, 5 mm) were cleaned in an ultrasonic cleaning bath with alkali solution and DI water and then dried using pure nitrogen. First, the output of power applied to the dual Cr targets was 4 kW in unipolar output mode. An interlayer of pure Cr was deposited at a DC bias of −120 V over a period of 5 min under a working pressure of 0.4 Pa. Then, the HiPIMS operating conditions were as follows: 5 kW in unipolar output mode, on-time for 150 μs (duty cycle of 3%) at a frequency of 200 Hz. The deposition duration of the AlCrN interlayer was 160 min under a working pressure of 0.4 Pa (N_2_:Ar flow = 160:160 sccm) under a DC bias of −60 V. The deposition time of AlCrCN films was fixed at 70 min. This research of AlCrCN coating was conducted in two parts: (1) assessing the effects of the C_2_H_2_ gas flow rate under a DC bias of −60 V and (2) assessing the effects of substrate bias voltage (−40 to −120 V) with the C_2_H_2_ flow rate fixed at 5 sccm. The working pressure was maintained at roughly 0.4 Pa through the injection of a gas mixture comprising Ar (160 sccm), N_2_ (160 sccm), and C_2_H_2_ (5, 8, 10, 13, 15, or 20 sccm). The rotating speed of the substrate holder was 3 rpm at a temperature of 200 °C. The crystalline structure of the AlCrCN layers was characterized using a grazing incidence X-ray diffractometer (GIXRD, PANalytical, X’pert MRD, Almelo, The Netherlands) with an incident angle of 0.5. The morphologies of the AlCrCN was characterized using a transmission electron microscopy (TEM, JEOL JEM-2100F, Tokyo, Japan). The TEM was operated at an accelerating voltage of 200 kV. The chemical compositions and bonding states were characterized using field-emission electron probe microanalyzer (FE-EPMA, JEOL, JXA-8500F, Tokyo, Japan) and X−ray photoelectron spectroscopy (XPS, ULVAC-PHI. Inc., PHI 5000 VersaProbe III, Kanagawa, Japan). The hardness and Young’s modulus were detected using a nanoindentation (TI-900, TriboIndenter, Hysitron, Minneapolis, MN, USA) with a Berkovich diamond probe tip. The loading rate was 1000 µN/s and the maximum indentation depth of 70–100 nm was controlled to minimize the influence of the interlayer and substrate on the hardness measurements. Each coating was measured ten times and the mean hardness was derived from the eight values that remained after removing the highest and lowest values. Rockwell indentation (C tip, 200 μm radius) tests were carried out under a 150 kg load for 10 s.

## 3. Results and Discussion

**Figure 1:**
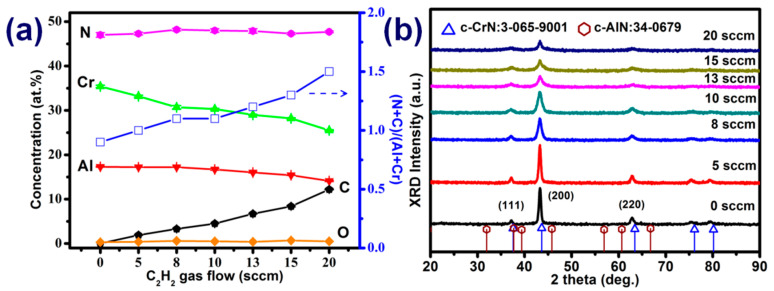
(**a**) Element concentrations and (**b**) XRD profiles of AlCrCN films deposited under various C_2_H_2_ flow rates.

Figure 1a illustrates the concentration of elements in the AlCrCN films deposited under various C_2_H_2_ flow rates. Increasing the C_2_H_2_ flow rate from 5 to 20 sccm increased the carbon content from 1.9 to 12.2 at.% and the (N + C)/(Cr + Al) ratio from 0.9 to 1.5. We did not observe saturation in terms of carbon content. The oxygen content in all films was less than 0.7 at.%. Increasing the C_2_H_2_ flow rate from 0 to 20 sccm changed the Cr:Al ratio from 3.0:7.0 to 3.5:6.5. The ratio of nitrogen to metal was used to determine the stoichiometry of the compound film. In a previous study, we demonstrated that the mechanical properties of stoichiometric compounds are generally superior to those of nonstoichiometric compounds [14]. In the current study, the (N + C)/(Cr + Al) ratio of the AlCrCN film exceeded 1.0. Note that Hu et al. [19] claimed that amorphous carbon bonds and the weak bonds of Cr-C are disadvantageous to the mechanical properties of AlCrCN coatings. This issue is discussed later in the context of XPS analysis. Figure 1b presents the XRD profile of AlCrCN films synthesized under various C_2_H_2_ gas flow rates. All of the observed diffraction peaks corresponded to CrN in accordance with JCPDS-ICDD 03-065-9001. Figure 1b presents no hcp-AlN or other compound phases, due to the fact that the Al content was below 70%. As the C_2_H_2_ flow rate was increased, the diffraction peaks of CrN (111), CrN (200), and CrN (220) became weaker and broader, due to deteriorating crystallinity in the AlCrN coating [29]. Crystallite sizes in the AlCrCN coatings were estimated using the Sherrer formula [13]. Increasing the C_2_H_2_ flow rate from 5 to 20 sccm led to a corresponding decrease in crystallite size along the (200) planes from 16.21 to 7.86 nm. This also shifted the reflection corresponding to the AlCrCN phase to an angle lower than that of the AlCrN phase, due to compressive stress imposed by ion beam bombardment [30,31].

**Figure 2:**
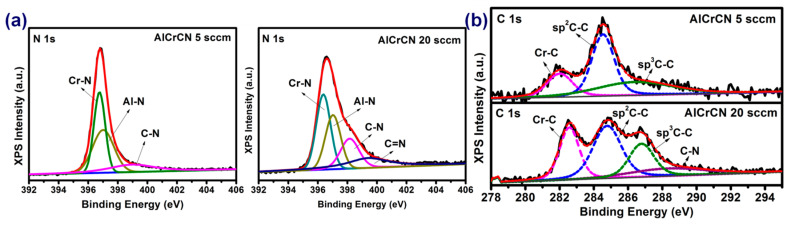
XPS spectra of AlCrCN film after deconvolution: (**a**) N 1s and (**b**) C 1s.

The chemical bonds in AlCrCN films were characterized using XPS analysis, the results of which are shown in Figure 2. The N_1s_ peaks in Figure 2a can be deconvoluted into four peaks (centered at): Cr-N (396.75 eV), Al-N (397.02 eV), C-N (398.2 eV), and C=N (400.01 eV) [20,32]. The C_1s_ peaks in Figure 2b were deconvoluted into four peaks (centered at): Cr-C (282.6 eV), sp^2^ (284.77 eV), sp^3^ (286.75 eV), C-N (288.3 eV) [20,33,34]. The fraction of each bond was calculated according to the individual area, the results of which are listed in Table 1. Increasing the C_2_H_2_ flow rate from 5 to 20 sccm led to the formation of C=N double bonds, while increasing the ratio of C-N bonds beyond that of metal-N bonds. The C_1s_ spectra revealed two main chemical bonds: Cr-C and sp^2^(a-C). In samples fabricated using the C_2_H_2_ flow rate of 5 sccm, we observed higher sp^3^/sp^2^ value (0.73) associated with AlCrCN films of higher hardness (26.8 GPa). Increasing the C_2_H_2_ flow rate to 20 sccm decreased the sp^3^/sp^2^ value (0.56) and metal-N content in the AlCrCN films, which led to a corresponding decrease in hardness (13.5 GPa). Researchers have previously reported that hardness is correlated with the sp^3^ fraction [20,35]. It has also been reported that the hardness of Cr-C compounds exceeds that of Cr-N compounds due to the formation of hard Cr_7_C_3_ phase [15]. However, we obtained no evidence of Cr_7_C_3_ phase in AlCrCN films fabricated under a C_2_H_2_ flow rate of 20 sccm in the current study.

**Figure 3:**
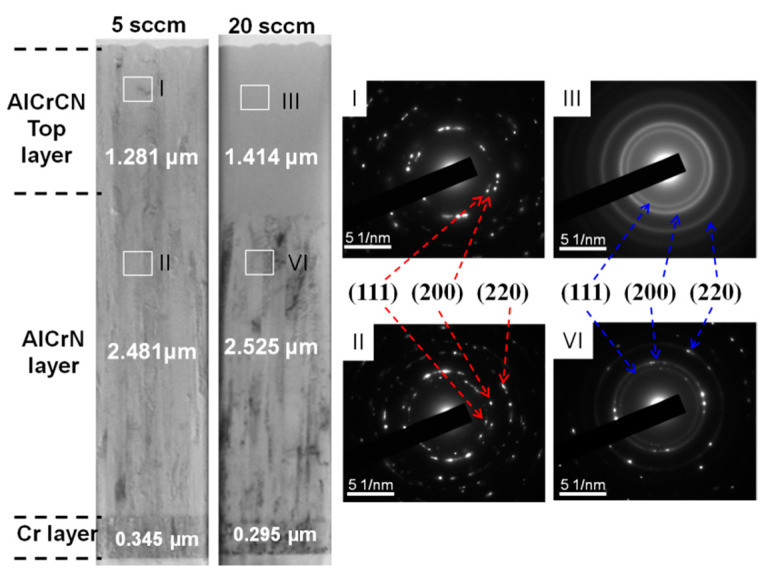
TEM image and SAED patterns of AlCrCN coatings as a function of C_2_H_2_ flow (5 and 20 sccm).

**Figure 4:**
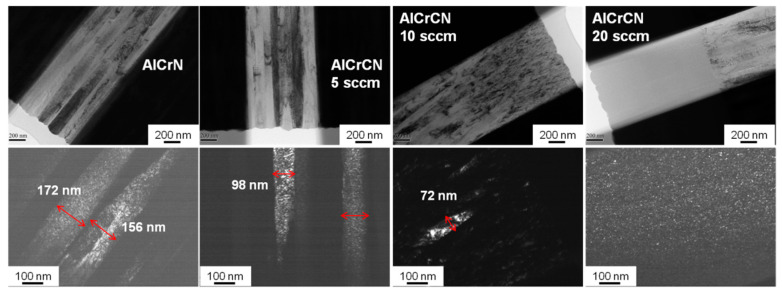
BF and DF TEM images of AlCrN and AlCrCN films.

Figure 3 presents the various hard coating designs and corresponding TEM results, illustrating the structure of the AlCrCN films as a function of C_2_H_2_ flow rate (5 or 20 sccm). Based on the TEM images, the structure of the coatings (total thickness of roughly 4 μm) can be divided into three layers (AlCrCN, AlCrN, Cr). Typical SAED patterns revealed that the AlCrN and AlCrCN films were polycrystalline, corresponding to (111), (200), and (220) crystalline planes of the FCC structure. As the C_2_H_2_ flow rate was increased, we observed a transformation in the diffraction patterns from diffraction spots to polycrystalline diffraction rings with the same radius. Figure 4 illustrates bright-field (BF) and dark-field (DF) TEM images of the FIB lamella, showing the microstructure of AlCrN and AlCrCN as a function of C_2_H_2_ flow rate (5 or 20 sccm). BF TEM images revealed the disappearance of the columnar structure in the top layer of AlCrCN as the C_2_H_2_ flow rate was increased. This can be attributed to C-N bonds inhibiting the development of metal-N bonds, leading to the formation of nanocrystallites in the AlCrCN film. DF TEM images revealed a decrease in columnar crystal size from 172 nm to the nanocrystalline scale as the C_2_H_2_ flow was increased from 0 to 20 sccm.

**Figure 5:**
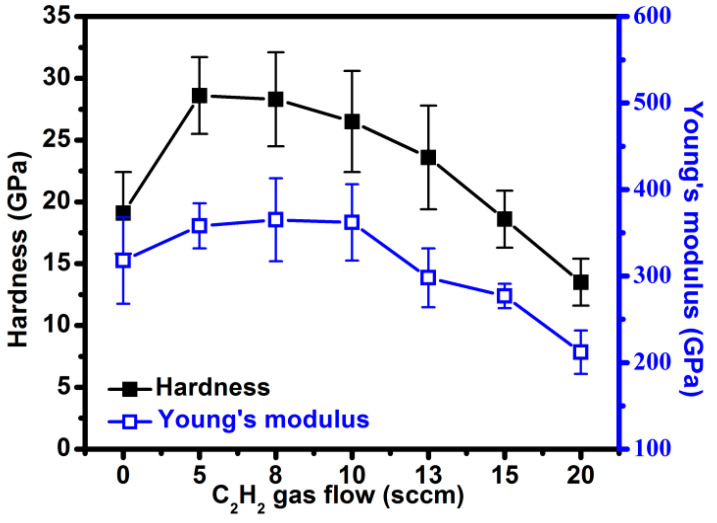
Nanoindentation results (hardness and Young’s modulus) as a function of C_2_H_2_ gas flow rate.

Figure 5 presents the hardness and Young’s modulus of AlCrCN films as a function of carbon content, as determined using nanoindentation measurements. When the C_2_H_2_ flow rate was increased from 0 to 20 sccm, we observed an initial increase in the hardness (from 19.1 to 28.6 GPa) and Young’s modulus (318 to 358 GPa), both of which subsequently decreased to 13.5 GPa and 212 GPa, respectively. The increased hardness of the AlCrN film can be attributed to a reduction in the size of the columnar crystals and grains, which restricted the displacement of dislocations, the propagation of cracks, and grain boundary sliding [8,36]. The AlCrCN film with the highest hardness presented fewer C-N and C=N bonds and a higher sp^3^/sp^2^ ratio. We can see in the TEM image that the columnar crystals in AlCrCN film deposited with a C_2_H_2_ flow rate of 10 sccm were smaller than that of films deposited under a flow rate of 5 sccm; however, the hardness was not as high. XPS results revealed that replacing a large number of metal−N bonds with C-N and C=N bonds resulted in an FCC structure with poor crystallinity. The main effect of hardness should come from the C-N bond inhibiting the development of metal-N bonds, leading to the formation of nanocrystallites in the AlCrCN film.

**Figure 6:**
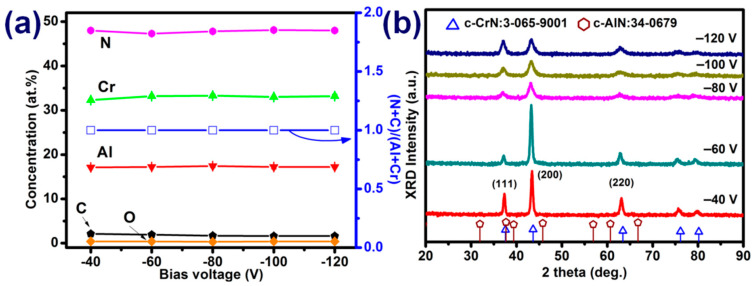
(**a**) Elements concentrations and (**b**) XRD profiles of AlCrCN films as a function of bias voltage.

Considering the H and E values and the error bars, the best and stable mechanical properties were obtained using a C_2_H_2_ flow rate of 5 sccm compared with the samples deposited at 8 and 10 sccm of C_2_H_2_. We therefore adopted this flow rate in subsequent experiments investigating the influence of bias voltage on the microstructure and hardness of AlCrCN films. The EPMA results in Figure 6a revealed that element content was unaffected by bias voltage. The XRD results in Figure 6b revealed a crystalline microstructure matching the FCC structure of CrN. Increasing the bias voltage from −40 to −120 V led to a decrease in crystallite size along the (200) plane (16.9, 16.2, 7.5, 7.3, and 7.1 nm).

**Figure 7:**
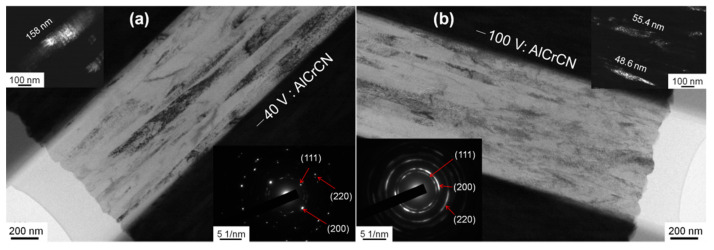
BF, SAED (**a** lower right and **b** lower left) and DF TEM (**a** top left and **b** top right) images of AlCrCN film as a function of bias voltage (−40 V and −100 V).

**Figure 8:**
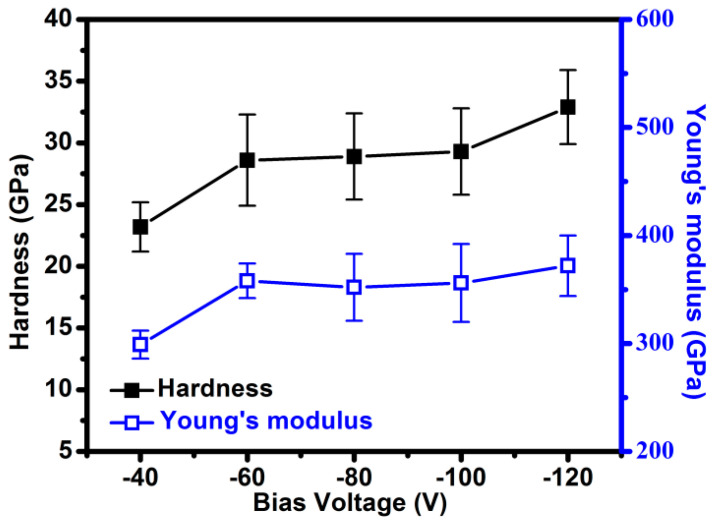
Nanoindentation results of AlCrCN films as a function of bias voltage.

**Figure 9:**
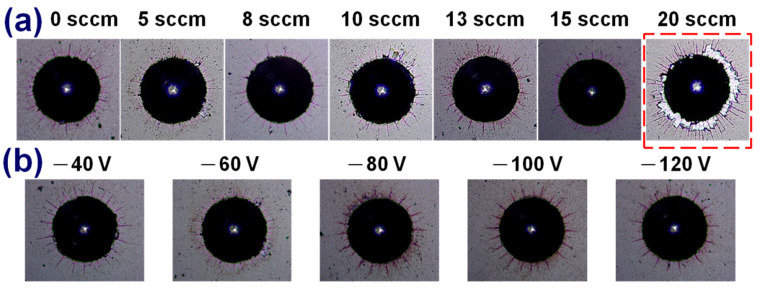
Rockwell indentation results of AlCrCN deposited as function of (**a**) C_2_H_2_ flow rate and (**b**) bias voltage.

The BF and DF images in Figure 7 revealed that under a bias voltage of −40 V, the columnar crystals in the CrAlCN layer were larger than those formed under a bias voltage of −100 V. Note that the SAED data were collected using an aperture with the same area. As the bias voltage was increased, the SAED patterns transformed from diffraction spots to polycrystalline diffraction rings, indicative of grain structure refinement [13]. Figure 8 presents the hardness and Young’s modulus of AlCrCN films as a function of bias voltage. Increasing the bias voltage to −120 V increased the hardness to 32.9 GPa and the Young’s modulus to 372 GPa. Note that the application of bias voltage to enhance hardness should also be applicable to carbon-doped AlCrN films as well. Figure 9 presents the results of Rockwell indentation tests performed on the AlCrN and AlCrCN films deposited on WC substrates in accordance with ISO26443:2008-06 standards, which is used to classify adhesive strength from high (class 0) to low (class 3). All of the samples in the current study fell within class 1, except for the samples deposited using a C_2_H_2_ flow rate of 20 sccm. Note that the poor adhesion properties of that sample can be attributed to decreased hardness and increases in the number of weak C-N and C=N bonds.

## 4. Conclusions

Our objective in this work was to elucidate the effects of C_2_H_2_ flow rate and DC bias voltage on the properties of C-doped AlCrN coatings deposited using HiPIMS. Increasing the C_2_H_2_ flow rate from 0 to 20 sccm reduced the ratio of sp^3^/sp^2^ and the grain size, as well as causing a structural transformation from a columnar crystal to nanocomposite structure. The hardness properties of the AlCrCN coating were improved from 19.1 to 28.6 GPa by introducing the C_2_H_2_ flow rate of 5 sccm, due to the phenomenon of grain and columnar refinement. When increasing the C_2_H_2_ flow rate to 20 sccm, the hardness of AlCrCN coatings deteriorates due to the C-N bond inhibiting the development of metal-N bonds. TEM image and SAED results were used to elucidate the phenomenon of grain and columnar refinement. Increasing the bias voltage from −40 V to −120 V decreased the size of crystallites in the AlCrCN layer at fixed the C_2_H_2_ flow rate of 5 sccm, which led to a corresponding increase in hardness from 23.2 GPa to 32.9 GPa. It appears that the application of bias voltage to enhance hardness is also applicable to carbon-doped AlCrN films. All of the samples presented good adhesion characteristics (class 1).

## Figures and Tables

**Table 1:** Area fractions of various bonds of AlCrCN films based on N 1s and C 1s XPS results.

C_2_H_2_ Flow	Cr-N (%)	Al-N (%)	C-N (%)	C=N (%)	Cr-C (%)	sp^2^ (%)	sp^3^ (%)	N-C (%)	sp^3^/sp^2^
5 sccm	40	40	20	0	20.2	46	33.8	-	0.73
10 sccm	39.4	39	9.7	11.9	21.4	30.4	20.4	27.8	0.67
20 sccm	34	27	19	20	26	38	21.2	14.8	0.56

## Data Availability

Not applicable.
